# Vaginal metabolome: towards a minimally invasive diagnosis of microbial invasion of the amniotic cavity in women with preterm labor

**DOI:** 10.1038/s41598-020-62542-6

**Published:** 2020-03-25

**Authors:** Sara Vicente-Muñoz, Teresa Cobo, Leonor Puchades-Carrasco, Ana B. Sánchez-García, Núria Agustí, Montse Palacio, Antonio Pineda-Lucena, Eduard Gratacós

**Affiliations:** 1 NMR-based Metabolomics Core, Division of Pathology and Laboratory Medicine, Cincinnati Children’s Hospital Medical Center, 3333 Burnet Avenue, Cincinnati, OH 45229 USA; 2 Hospital Clinic of Barcelona, BCNatal - Barcelona Center for Maternal-Fetal and Neonatal Medicine (Hospital Clínic and Hospital Sant Joan de Déu), Fetal i + D Fetal Medicine Research Center, Consorci Institut d’Investigacions Agustí Pi I Sunyer (IDIBAPS), University of Barcelona, Barcelona, Spain; 3 Centre for Biomedical Network Research on Rare Diseases (CIBER-ER), Barcelona, Spain; 4 Drug Discovery Unit, Instituto de Investigación Sanitaria La Fe, Hospital Universitario y Politécnico La Fe, Valencia, Spain; 5 Structural Biochemistry Laboratory, Centro de Investigación Príncipe Felipe, Valencia, Spain; 6 Molecular Therapeutics Program, Center for Applied Medical Research, University of Navarra, Pamplona, Spain; 7 Present Address: APL: Medicinal Chemistry Laboratory, Molecular Therapeutics Program, Center for Applied Medical Research, University of Navarra, Pamplona, Spain

**Keywords:** Biomarkers, Medical research, Molecular medicine

## Abstract

Microbial invasion of the amniotic cavity (MIAC) is only identified by amniocentesis, an invasive procedure that limits its clinical translation. Here, we aimed to evaluate whether the vaginal metabolome discriminates the presence/absence of MIAC in women with preterm labor (PTL) and intact membranes. We conducted a case-control study in women with symptoms of PTL below 34 weeks who underwent amniocentesis to discard MIAC. MIAC was defined as amniotic fluid positive for microorganisms identified by specific culture media. The cohort included 16 women with MIAC and 16 control (no MIAC). Both groups were matched for age and gestational age at admission. Vaginal fluid samples were collected shortly after amniocentesis. Metabolic profiles were analyzed by nuclear magnetic resonance (NMR) spectroscopy and compared using multivariate and univariate statistical analyses to identify significant differences between the two groups. The vaginal metabolomics profile of MIAC showed higher concentrations of hypoxanthine, proline, choline and acetylcholine and decreased concentrations of phenylalanine, glutamine, isoleucine, leucine and glycerophosphocholine. In conclusion, metabolic changes in the NMR-based vaginal metabolic profile are able to discriminate the presence/absence of MIAC in women with PTL and intact membranes. These metabolic changes might be indicative of enhanced glycolysis triggered by hypoxia conditions as a consequence of bacterial infection, thus explaining the utilization of alternative energy sources in an attempt to replenish glucose.

## Introduction

Maternal and fetal medicine is moving toward individualized patient-care aiming to identify the most appropriate clinical management for each woman. In women with preterm labor (PTL), early spontaneous preterm deliveries (PTD) are more likely related to microbial invasion of the amniotic cavity (MIAC) and intra-amniotic inflammation (IAI)^1^. Diagnosis of MIAC help clinicians to identify women with high-risk of delivering in the following days^2^. This information may be used to efficiently plan antenatal management^3^ by transfer to facilities with Neonatal Intensive Care Units (NICU), administration of antenatal steroids^4^, magnesium sulfate^5^ and, probably, with specific antibiotics^6^ according to the microorganism isolated. Furthermore, additional information about MIAC may not only render antenatal strategies, such as tocolysis, questionable, but may also help neonatologists improve neonatal management.

Nowadays, amniocentesis is the only procedure to identify MIAC since it can occur without a clinical suspicion. Despite the very low rate of complications associated with amniocentesis^7^, the invasive nature of this procedure limits its clinical translation. This limitation has led to exploring the intra-amniotic environment using minimally invasive strategies based on the analysis of different biofluids, including vaginal fluid^8^.

In this context, high-dimensional biology referred to as “omics” provide a comprehensive description of the biological processes and has facilitated better understanding of the biochemical changes associated with complex diseases, and is able to identify biomarkers and advance in translational research. Metabolomics appears to be one of the most promising approaches in maternal and fetal medicine to study the complex interactions between mother, placenta, and fetus^9^. It allows simultaneous examination and relative quantification of changes in the metabolome, which is the final downstream product of all the biochemical processes and more closely reflects the phenotype at a functional level. Furthermore, metabolomics allows the understanding of the molecular mechanisms responsible for the deregulation and provides a logical strategy for intervening in the process^10^. Nuclear Magnetic resonance (NMR) spectroscopy is a profiling technology that provides highly reproducible information on complex biological fluids or tissues, with minimal preparation and in an essentially non-destructive manner.

Several metabolomic studies have monitored healthy pregnancy^11,12^. Other reports have mainly focused on selected stages or disorders of pregnancy, such as fetal malformations^13^, gestational diabetes mellitus^14^, preeclampsia^15^, neural tube defects^16^, very-low birth weight^17^, fetal lung maturity^18^ and PTD^19–25^. Regarding MIAC or IAI, Romero *et al*.^20^ found differences in the amniotic fluid metabolome in women with symptoms of PTL according to information of MIAC. Brown *et al*.^26^ observed changes in the metabolic profiles of amniotic fluid, fetal brain and postnatal brain of pregnant mice exposed to IAI. However, to our knowledge, there are no data on the metabolome profile of women with PTL to diagnose MIAC using a minimally invasive approach.

This study describes an untargeted ^1^H-NMR metabolomics approach, based on the analysis of vaginal fluid to identify metabolic profiles associated with MIAC. The characterization of this minimally invasive biofluid provides information on the metabolic networks directly related to the presence of an ascending bacterial infection in the amniotic fluid and to explore the effect of the process through vaginal fluid. In addition, identification of a vaginal metabolic profile of MIAC in women with PTL and intact membranes. could help clinicians to target women who might benefit from amniocentesis for this indication. Therefore, the main objective of this study was to characterize the vaginal metabolome profile of MIAC in women with PTL presenting intact membranes.

## Results

This case-control study included 32 women, 16 in each group. Maternal clinical characteristics and demographic details of the women included in the study are summarized in Table 1. All women with PTL had intact membranes.

**Table 1:** Maternal characteristics and perinatal outcome.

	MIAC group (n = 16)	No-MIAC group–Control (n = 16)	*p*. value
Maternal age	33.4 (24.2; 37.6)	31.8 (27.0; 35.7)	0.386
Caucasian ethnicity	16 (100)	15 (93.8)	1.000
Nulliparity	8 (50)	8 (50)	1.000
Prior PTD	4 (25)	3 (18.8)	1.000
CRP (mg/L) at admission	3.8 (0.8;7)	0.6 (0.3; 1.3)	0.002
WBC (x10^9^/L) at admission	12280 (10077; 16652)	12635 (9832; 14600)	0.546
US CL (mm) at admission	3 (0; 25)	15 (8; 23)	0.097
Positive genital cultures at admission	8 (50)	3 (18.8)	0.135
GA at sampling (weeks)	25.0 (22.3; 30.3)	26.9 (26.3; 30.8)	0.169
AF glucose (mg/d)	4 (1.2; 14)	33.5 (24.2; 38.7)	<0.0001
Steroid administration	13 (81.2)	15 (93.8)	0.600
Antibiotic treatment	16 (100)	5 (31.2)	<0.0001
Antibiotic treatment prior to vaginal sampling (days)	0 (0; 0)	0 (−0.5; 30.5)	0.839
Tocolysis treatment	14 (87.5)	15 (93.8)	1.000
GA at delivery (weeks)	26.3 (25.1; 30.5)	37.1 (34.1; 39.9)	<0.0001
Latency from sampling to delivery (days)	2.5 (1; 5.7)	63 (40.2; 79.7)	<0.0001
Birth weight (g)	870 (667.5; 1363.7)	2894 (1985; 3407.5)	<0.0001
Male gender	6 (37.5)	7 (43.8)	1.000

MIAC: Microbial invasion of the amniotic cavity; PTD: Preterm delivery; CRP: C-reactive protein; WBC: White blood cells; US CL: Ultrasound cervical length; GA: Gestational age; AF: Amniotic fluid. Continuous variables were compared using a nonparametric Mann-Whitney U test presented as medians (25^th^; 75^th^ percentile). Categorical variables were compared using Chi-square or Fisher exact test and presented as number (%). p value < 0.05 was considered statistically significant.

Sixteen women had MIAC. The microorganisms most frequently isolated in the amniotic fluid were *Ureaplasma* spp. (n = 5) and *Fusobacterium nucleatum* (n = 5). Other microorganisms isolated were *Listeria monocytogenes* (n = 1), *Streptococcus viridans* (n = 1), *Capnocytophaga sputigena* (n = 1), *Escherichia coli* (n = 1) and *Candida albicans* (n = 2). We did not find differences in the rate of positive vaginal cultures or in the antenatal management (e.g., antenatal steroids, tocolysis or antibiotics administration prior to vaginal sampling) between groups.

### ^1^H-NMR metabolic profile of vaginal samples

**Figure 1:**
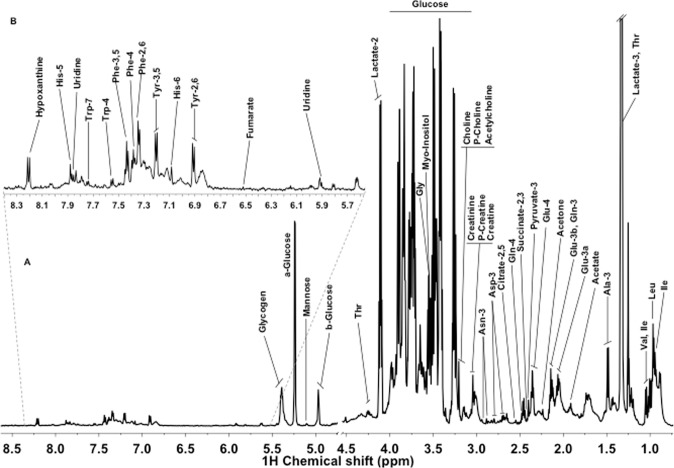
Representative 600-MHz ^1^H-NMR spectrum of a vaginal fluid sample from a woman with presence of MIAC. (**A**) Full spectrum (δ 8.5–0.5 ppm); **(B)** Magnification of the spectrum region from δ 5.6–8.4 ppm.

^1^H-NMR CPMG spectra were acquired for all the vaginal samples included in the study. Figure 1 shows a representative ^1^H-NMR vaginal spectrum from a woman with MIAC. A total of 34 metabolites were assigned in the region between 0.5 and 8.5 ppm, including amino acids (e.g., leucine, valine, alanine, threonine, glutamine, phenylalanine), organic acids (e.g. lactate, fumarate, formate), energy-related metabolites, sugars and lipids. Direct analysis by NMR was not possible due to extremely high viscosity of the vaginal fluid and therefore this biofluid was lyophilized and consequently reconstituted in 100% D_2_O. The quality of the spectra and signal-to-noise ratio obtained proved to be a robust method to analyze vaginal samples by NMR.

### Multivariate statistical analysis and group discrimination

For identifying the differences between both groups, multivariate statistical analysis was applied to all the samples included in the study. First, a non-supervised analysis on vaginal fluid ^1^H-NMR spectra was performed for identifying intra- and inter-groups variability. A PCA was carried out to evaluate sample homogeneity within each group of samples and examine the potential influence of different clinical variables (i.e., gestational age at admission, days between admission and delivery) in the overall distribution of the data. PCA analysis did not reveal any pattern or clustering of the samples according to different clinical variables (i.e., gestational age at admission, days between admission and delivery) (data not shown). However, this analysis did not reveal either any pattern or clustering of the samples according to these descriptors; thus, it was possible to discard these variables as potential confounding factors. Furthermore, dilution differences were identified between vaginal samples, probably derived from the method of sample collection. This variability in sample concentration did not correlate with respond clinical variable or condition, probably reflecting inter-individual variability in vaginal fluid concentration. This effect was compensated using PQN normalization in vaginal samples.

Next, OPLS-DA method was applied, as method of classification, to compare the two groups of patients included in the study and identify the differences associated with these clinical situations. The samples were classified as positive (+AF) or negative (−AF) based of the presence or absence of MIAC, respectively. The score plot showed a clear separation between vaginal samples collected from women with and without MIAC (R^2^Y = 0.525 and Q^2^ = 0.296) (data not shown).

**Figure 2:**
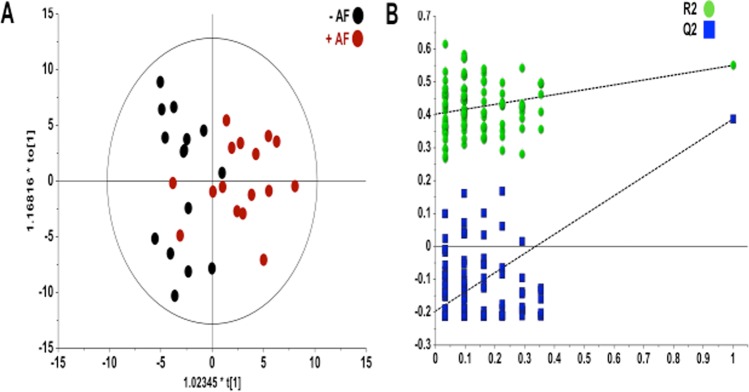
Multivariate Statistical model resulting from the analysis of ^1^H-CPMG spectra for the comparison between control individual (red) vs. women with MIAC (black) and the corresponding internal cross-validation test. (**A**) OPLS-DA score plot calculated after variable selection (R^2^Y(cum): 0.535; Q^2^(cum): 0.405) showing the separation between both groups of samples. (**B**) Permutation test (n = 100) obtained from the corresponding PLS-DA model with an equivalent number of components used for the OPLS-DA model with values of intercepts R^2^: 0.402 and Q^2^: −0.197.

To select the most relevant variables contributing to the discrimination of the women with and without MIAC, variable selection was performed using the ratio between regression coefficients values and the Jack-knife standard error of their coefficients (CoeffCS/CoeffCScvSE). These ratio values were used to select those variables, which mainly contribute to the separation between both groups of samples. Applying this strategy, only those variables with a ratio larger than 1 were maintained and used to build the new OPLS-DA model. The rest of the descriptors were excluded as they were deemed uninformative variables with low or null discrimination ability in the model; therefore, not correlated with biological differences associated with the disease. After variable selection, a new OPLS-DA model (Fig. 2A) was built with the subset of selected variables. In the new model both goodness of fit and prediction were improved (R^2^Y = 0.535 and Q^2^ = 0.405) exhibiting a clear separation between the two groups of samples. The quality of the OPLS-DA obtained was validated with a permutation test. After one hundred class permutations (Fig. 2B) the validation plot presented R^2^ and Q^2^-intercept values were significantly lower than the original model.

### Characterization and quantification of the infection metabolic profile

Multivariate data analysis facilitated the identification of variables responsible for the separation between the two groups. Each relevant region, in terms of VIP values, was subsequently evaluated individually. Thus, a total of 52 buckets with VIP value >1 were obtained based on the analysis of the final OPLS-DA model. These spectral regions were considered very relevant variables in the separation between the two groups (No-MIAC *vs*. MIAC).

Univariate statistical analysis of the previously selected spectral regions revealed significant differences (*p*-value < 0.05) between the two groups. The levels of four metabolites were found to be significantly higher in the vaginal fluid from women with MIAC (hypoxanthine (85.90%), proline (88.83%), choline (71.56%) and acetylcholine (30.96%)), and five metabolite levels were lower as compared to control women (phenylalanine (−35.14%), glutamine (−29.66%), leucine (−33.69%), isoleucine (−27.63%) and glycerophosphocholine (−32.85%)) (Table 2). Benjamini-Hochberg adjusted *p*-values are shown in Supplementary Table 1.

**Table 2:** Variations for the statistically significant metabolites involved in the discrimination between women with MIAC (n = 16) and without (n = 16).

δ ^1^H (ppm)^a^	Metabolite(s)	*p*. value^b^	% Variation^c^
8.1845–8.1715	Hypoxanthine	0.001	85.90
7.3845–7.3425	Phenylalanine	0.032	−35.14
3.3405–3.3315	Proline	0.003	88.83
3.2135–3.2045	Glycerophosphocholine	0.007	−32.85
3.2035–3.1955	Acetylcholine	0.042	30.96
3.1945–3.1795	Choline	0.007	71.56
2.3515–2.3105	Glutamine	0.004	−29.66
1.0035–0.9914	Isoleucine	0.046	−27.63
0.9604–0.9374	Leucine	0.035	−33.69

^a^Chemical shift range for the integration; ^b^p. value calculated by Wilcoxon-Mann-Whitney U test; ^c^relative variation of the metabolite levels in women with MIAC compared with women without MIAC.

## Discussion

Our preliminary data, derived from the analysis of metabolite profiles using multivariate and univariate statistical methods, revealed that the vaginal metabolic profile of women with PTL and intact membranes discriminates between women with and without MIAC.

Previous studies have shown vaginal metabolome profile related to the occurrence of bacterial vaginosis (BV) and other clinical outcomes such as PTD. Thus, an untargeted metabolomics analysis of vaginal fluid identified biomarkers associated with BV and validated the results in a blinded replication cohort^27^ with 91% accuracy. A recent study reported the metabolic profile of women with and without BV using mass spectrometry-based metabolomics and classified women based on their vaginal metabolite composition^28^. Amabebe *et al*.^29^ hypothesized that the cervicovaginal ecosystem of commensal and pathogenic organisms could induce a specific metabolic profile that could be used for predicting the risk of PTD. In agreement with their previous report^29^, they demonstrated the potential of cervicovaginal acetate levels as a predictor of PTD using ^1^H-NMR spectroscopy, especially in women with symptoms of PTL^30^.

Our study focused on the characterization of the vaginal metabolome of women with MIAC. The majority of MIAC have an ascending origin^31^. Vaginal secretions from the women recruited in this study exhibited specific metabolite changes associated with infection. Their vaginal metabolic profile showed decreased concentrations of amino acids (phenylalanine, glutamine), branched-chain amino acids (leucine, isoleucine), and glycerophosphocholine as well as increased hypoxanthine, proline, choline and acetylcholine concentrations (Table 2).

It is known that bacterial pathogens trigger a specific host response characterized by: (a) the induction of host cell enzymes, which dampens the production or action of reactive oxygen species (ROS) and reactive nitrogen intermediates, (b) a further enhanced glucose uptake and glycolysis which stimulate the anabolic activity of the host cells (e.g., by increased production of nucleotides and amino acids), and (c) a switch to enhance glutaminolysis and citrate lyase reaction reinforcing fatty acid/lipid biosynthesis^32^.

One of the most significant differences is associated with the levels of free choline, being much higher in vaginal samples from women with MIAC. During gestation, there is a high demand for this essential nutrient^33^. Large amounts of choline are delivered to the fetus across the placenta^34^. Choline is closely associated with fetal brain development and has also been shown to change in relation to hypoxia and pre-eclampsia^35,36^. Werth and co-workers demonstrated that hypoxia inducible transcription factor-1 (HIF-1) activation in infections with human pathogenic microorganisms is a general phenomenon^37^. Therefore, it would likely explain the increased levels of choline observed, as well as those from its downstream product acetylcholine. Hypoxanthine, an ATP degradation product under hypoxic conditions^36^ has been defined as biomarkers of hypoxia, hypoxemia and ischemic brain injury^38^. Elevation of hypoxanthine has also been reported during septic shock and may reflect early high energy nucleotide failure^39^. Moreover, circulating hypoxanthine levels have been found to increase during the febrile phase^40^, as well as in models of chronic systemic inflammation^40^. Based on these previous findings, it is feasible that similar events could be observed in infectious diseases in which acute bouts of inflammation occur, such as in MIAC.

The vaginal profile of women with MIAC exhibits a profound disturbance in amino acid metabolism that is characterized by decreased levels of phenylalanine, isoleucine, glutamine and leucine. This finding could be consistent with an increased glycolysis induced by hypoxic conditions, thus explaining the utilization of selected amino acids as an alternative energy source for the replenishing of glucose^41^. To this end, it is important to point out that short peptides are usually taken by microorganisms, for optimal growth during infection, and degraded into amino acids, that are eventually catabolized for replenishing TCA cycle intermediates and generating gluconeogenic substrates^42^.

Taken together, the vaginal metabolic profile observed in women with MIAC seems to reflect the interplay between microorganisms and host and involves a large variety of physiological responses. One of the strengths of the study is the well-characterized study population with information of MIAC, the accurate sampling processing and the close perinatal follow-up. Some of the limitations of this study refer to the method used for detecting MIAC, we did not perform genomic non-cultivation technology analyses. However, to date, amniotic fluid cultures continue to be the “gold standard” to define MIAC in most clinical settings. Furthermore, we acknowledge the small number of samples available from MIAC women presenting PTL and intact membranes. These preliminary data require to be validated in a larger cohort to ensure reproducibility; special efforts will be paid to the collection of an independent validation cohort. Finally, we did not characterize vaginal microbiota in these women being therefore unable to investigate the relation between vaginal microbiota and metabolome expression in women with MIAC. In this regard, some studies have shown how vaginal microbiome modulates metabolome expression in women with genital infections^43–45^. Future studies evaluating the association between vaginal microbiota, metabolomic profiles and perinatal outcomes, such as spontaneous preterm birth, in women with MIAC are warranted.

In conclusion, a specific vaginal metabolome profile of MIAC was characterized in women with PTL and intact membranes. The results of this study open the possibility to target women with a high-risk of MIAC using minimally invasive sampling method, thereby limiting amniocentesis in this high-risk group. In addition, pending validation, this experimental setup could be expanded to the evaluation and monitoring of the infectious process and, eventually, the response to specific therapies in women with MIAC.

## Materials and Methods

### Study design and subject recruitment

This study was conducted as a case-control study and included a group of 32 women with symptoms of PTL and intact membranes admitted to the Maternal and Fetal Medicine Department of Hospital Clinic, Barcelona, under 34 weeks of gestation with an amniocentesis to discard MIAC. Both groups were matched for age and gestational age at admission. Clinical diagnosis and classification of women was performed according to the presence/absence of MIAC. Exclusion criteria were clinical signs of chorioamnionitis, multiple gestation, preterm prelabor rupture of membranes, fetuses with aneuploidy, maternal diabetes, women who did not consent to participate in the study, and cases in which amniocentesis was not technically possible.

Gestational age was established according to the first-trimester ultrasound scan. PTL was defined as the presence of regular uterine contractions with a frequency of at least 2 every 10 minutes and an ultrasound cervical length below the 5^th^ percentile for gestational age^46^. MIAC was defined as a positive aerobic/anaerobic amniotic fluid culture for bacteria or yeast or the presence of *Ureaplasma* spp. or *Mycoplasma hominis* in genital mycoplasma culture.

Study population recruitment and sample collection were carried out at the Materno-fetal Medicine Department of Hospital Clinic, Barcelona (Spain) from 2010 to 2013. NMR spectra acquisition and the metabolomics analysis were performed at *Centro de Investigación Príncipe Felipe*, Valencia (Spain).

### Ethical approval

Recruitment and sampling procedures were performed in accordance with the Declaration of Helsinki and applicable local regulatory requirements after approval from the Ethics Committee of the Hospital Clinic of Barcelona (HCB/2010/5811). Written informed consent was obtained from all the subjects included in this study.

### Clinical management

The antenatal management of women with PTL has been reported previously^47^ and includes fetal maturation with a complete course of antenatal steroids (betamethasone) between 24.0 to 34.6 weeks and tocolysis (nifedipine or atosiban). Antibiotics (ampicillin, gentamycin, azithromycin) were prophylactically initiated in women with advanced cervical dilatation (Bishop index >6) and were discontinued if the amniotic fluid culture result was negative. Women with amniotic fluid glucose levels <5 mg/dL and/or with microorganisms on Gram staining and/or positive amniotic fluid cultures were treated with antibiotics according to the antibiogram of microorganisms isolated.

### Amniotic and vaginal fluid collection

Amniotic fluid samples were obtained by transabdominal amniocentesis at admission. Cultures for genital mycoplasma (Mycoplasma IST 2, bioMérieux for *Ureaplasma* spp. or *Mycoplasma hominis)*, aerobic (Chocolate agar), anaerobic (Schaedler agar for anaerobes and thioglycollate broth) bacteria, as well as amniotic fluid glucose concentrations and Gram stains were performed immediately after the procedure and the results were available for clinical decisions.

Vaginal fluid samples were collected using Cytobrush swabs (Cytobrush Plus GT; Medscan Medical AB) from the posterior vaginal fornix shortly or within 24–48 h after amniocentesis sampling. Each Cytobrush was submerged in 1.0 mL of sodium chloride (NaCl) (9 mg/mL) and kept at 4 °C until processing. Vaginal fluid was centrifuged at 3421 *g* at 4 °C for 10 minutes and supernatant was stored at −80 °C until the NMR analysis.

### NMR sample preparation

Vaginal fluid samples were thawed on ice and gently mixed before sample preparation. 500 µL of freeze-dried vaginal fluid sample were dissolved in 500 µL of D_2_O containing 1 mM of trimethylsilylpropionic acid-d4 sodium salt (TSP) as internal standard compound^48,49^, and 60 µL of 1.5 M potassium phosphate buffer (pH 7.4) in D_2_O. The mixture was transferred to a 5-mm and stored at 4 °C until analysis.

### ^1^H-NMR Spectroscopy of vaginal fluid

#### NMR data acquisition

NMR spectra were acquired using a Bruker Avance II 600 MHz spectrometer (Bruker Biospin, Rheinstetten, Germany) equipped with a triple resonance cryo-probe with a cooled ^13^C preamplifier (TCI). ^1^H-NMR experiments were acquired at 310 K using a standard Carr-Purcell-Meiboom-Gill (CPMG) spin-echo pulse sequence^50^, which generates spectra edited with reduced broad resonances from high molecular weight improving the resolution of low molecular weight metabolites resonances. For each sample, 128 transients were collected into 74 K data points using a spectral width of 20 ppm. Water resonance was presaturated with a 25-Hz pulse during a 4-second relaxation delay. Free induction decays were multiplied by an exponential function equivalent to a 1.0 Hz line-broadening factor before applying Fourier transformation. Additionally, 2D ^1^H-^1^H TOtal Correlation SpectroscopY (TOCSY) and 2D ^1^H, ^13^C Heteronuclear Single Quantum Correlation (HSQC) experiments were performed for selected samples, to facilitate the identification of biochemical substances. Most of the metabolites were assigned comparing vaginal profiles with compounds included in the BBIOCODEREF library (Bruker BioSpin) on Analysis of Mixture software (v. 3.9.7; Bruker BioSpin). This information was complemented with other existing public databases HMDB (Human Metabolome database) and BMRB (Biological Magnetic Resonance Data Bank) and literature reports^51,52^.

### Spectral data processing

All transformed 1D ^1^H-CPMG spectra were phased and baseline corrected automatically using TopSpin (v 2.1, Bruker BioSpin, Rheinstetten, Germany). Spectra were internally calibrated with reference to the CH_3_ alanine peak at 1.47 ppm. For spectral data reduction, each vaginal spectrum (δ 0.60–8.46) was segmented into consecutive equal width buckets (0.04 ppm) using AMIX software (v 3.9.7; Bruker BioSpin). After excluding residual water signal (δ 4.41–4.87) and baseline regions, the spectra were normalized by probabilistic quotient normalization (PQN)^53^ to compensate dilution differences in sample concentration.

### Multivariate statistical analysis

Analysis of metabolite profiles was carried out using SIMCA-P software (v.13.0.2, Umetrics). Prior to this analysis, data were scaled to unit variance (UV) scaling, where the variable is centered and scaled by dividing by its standard deviation, giving all of the variables equal importance. First, unsupervised principal component analysis (PCA) was performed for finding potential patterns, intrinsic clusters and outliers. Next, orthogonal partial least square to latent discriminant analysis (OPLS-DA), a supervised method, was applied for maximizing class discrimination and minimizing the possible contribution of intragroup variability. The OPLS-DA model was assessed by R^2^Y (quantitative measure of goodness of fit) and Q^2^ (predictive ability calculated by 7-fold internal cross-validation rounds) parameters.

The initial OPLS-DA model was improved using variable selection to identify the relevant predictors and to exclude uninformative variables. The centered and scaled regression coefficients (CoeffCS) and their Jack-knife standard error of the coefficients (CoeffCScvSE) computed from all rounds of cross validations were used to discard spectral regions that were redundant or not correlated to the response^54^. With this strategy, only those variables, with a ratio between CoeffCS and CoeffCScvSE >1, were included in the model. Once applied, the variable selection method, a new OPLS-DA model, was calculated and internal cross validation and permutation tests were performed to evaluate the model obtained. Variables or spectral regions (buckets) with variable importance in the projection (VIP) scores larger than 1 from the 7-fold cross validated OPLS-DA model were considered responsible for the discrimination between both groups and were evaluated individually by univariate statistical analysis as potential biomarkers.

Permutation tests (n = 100) were carried out using SIMCA-P software (version 13.0.2, Umetrics), as internal validation method, for the evaluation of the quality of the OPLS-DA obtained. The class label of each sample was randomly permuted, and R^2^Y and Q^2^ values obtained from the permutated models were compared with the R^2^Y and Q^2^ values from the original model.

### Univariate statistical analysis and metabolite quantification

Relevant spectral regions responsible for the discrimination between the two groups, as derived from the multivariate OPLS-DA models, were selected and evaluated independently. The relative concentration of each metabolite was calculated by integrating the signals in the spectra. For continuous variables statistical significance was assessed using the non-parametric Wilcoxon-Mann-Whitney U test, metabolites with a *p*. value < 0.05 were considered significantly different. Chi-square test was applied for categorical variables. A *p*. value < 0.05 with two-sided alternative hypotheses were considered statistically significant. Benjamini-Hochberg correction was performed to control the false discovery rate^55^.

## Supplementary information

Supplementary Information.
