# Supplementary material for: Vaginal metabolome: towards a minimally invasive diagnosis of microbial invasion of the amniotic cavity in women with preterm labor
Source: Sci Rep. 2020 Mar 25;10:5465. doi: 10.1038/s41598-020-62542-6 (PMC7096387; doi:10.1038/s41598-020-62542-6)
Supplement: Supplementary file 1 — Supplementary Information. [file 41598_2020_62542_MOESM1_ESM.docx]

**SUPPLEMENTARY MATERIAL**

**Vaginal metabolome: towards a minimally invasive diagnosis of microbial invasion of the amniotic cavity in women with preterm labor**

Sara Vicente-Muñoz, Ph.D.^1^; Teresa Cobo, M.D., Ph.D.^2,3^*; Leonor Puchades-Carrasco, Ph.D.^4^; Ana B Sanchez-Garcia, BSc^2^; Núria Agusti, M.D.^2^; Montse Palacio, M.D., Ph.D. ^2,3^; Antonio Pineda-Lucena, Ph.D.^4‡^ and Eduard Gratacós, M.D., Ph.D.^2,3^

^1^NMR-based Metabolomics Core, Division of Pathology and Laboratory Medicine, Cincinnati Children's Hospital Medical Center, 3333 Burnet Avenue, Cincinnati, OH 45229, USA.

^2^Hospital Clinic of Barcelona, BCNatal - Barcelona Center for Maternal-Fetal and Neonatal Medicine (Hospital Clínic and Hospital Sant Joan de Déu), Fetal i+D Fetal Medicine Research Center, IDIBAPS, University of Barcelona. Barcelona, Spain.

^3^Center for Biomedical Research on Rare Diseases (CIBER-ER). Barcelona, Spain.

^4^Structural Biochemistry Laboratory, Centro de Investigación Príncipe Felipe. Valencia, Spain.

**Supplementary Table 1: Variations for the statistically significant metabolites, and adjusted *p*-values, involved in the discrimination between women with MIAC (n = 16) and control women (n = 16)**

| **δ ^1^H (ppm)^a^** | **Metabolite** | ***p-*value^b^** | **% Variation^c^** | **Adjusted ^d^**  **BH *p*-value** |
| --- | --- | --- | --- | --- |
| 8.1845 - 8.1715 | Hypoxanthine | 0.001 | 85.90 | 0.006 |
| 7.3845 - 7.3425 | Phenylalanine | 0.032 | -35.14 | 0.031 |
| 3.3405 - 3.3315 | Proline | 0.003 | 88.83 | 0.012 |
| 3.2135 - 3.2045 | Glycerophosphocholine | 0.007 | -32.85 | 0.025 |
| 3.2035 - 3.1955 | Acetylcholine | 0.042 | 30.96 | 0.043 |
| 3.1945 - 3.1795 | Choline | 0.007 | 71.56 | 0.025 |
| 2.3515 - 2.3105 | Glutamine | 0.004 | -29.66 | 0.018 |
| 1.0035 - 0.9914 | Isoleucine | 0.046 | -27.63 | 0.05 |
| 0.9604 - 0.9374 | Leucine | 0.035 | -33.69 | 0.038 |

^a^ Chemical shift range for the integration; ^b^ p. value calculated by Wilcoxon-Mann-Whitney U test; ^c^ relative variation of the metabolite levels in women with MIAC compared with women without MIAC. BH: Benjamine-Hochberg method using FDR 0.05.
